# Application of a hand-held supplementary light for extending field-based net ecosystem exchange carbon flux measurements in low light conditions

**DOI:** 10.1186/s40543-025-00496-y

**Published:** 2025-08-04

**Authors:** Andreas Heinemeyer, Thomas Holmes, Anthony Jones, Bing Liu, Jason Daff

**Affiliations:** 1 University of York, Stockholm Environment Institute (York Centre), Wentworth Way, Heslington, York, YO10 5NG UK; 2 University of York, Department of Biology, Wentworth Way, Heslington, York, YO10 5DD UK

**Keywords:** Carbon cycling, Flux chamber, Light response curve, Light condition, Seasonality

## Abstract

Manual chamber-based carbon flux measurements are frequently used to capture terrestrial carbon cycle processes over vegetated areas. Light response curves, achieved by sequential shading, enable obtaining model parameters of light compensation points, maximum photosynthetic rates and dark respiration. However, light conditions in the field are sometimes, or in higher northern latitudes frequently limited, especially in darker seasons and areas with frequent cloud and fog, which questions the robustness of fitted model parameters. Artificial light therefore offers a crucial way to assess and address these potential limitations, especially recent advances in LED lights with improved wavelength spectra and irradiance. However, previous LED lights were fixed on the chamber top, blocking out natural light, heavy and with a high-power demand unsuitable for remote field deployment. Here we tested a handheld LED torch as a flexible, low-power and low-weight option. We investigated the wavelength spectrum and photosynthetically active radiation (PAR) output under controlled conditions and applied it under light limiting field conditions. Increased PAR from short-term measurements did not increase chamber temperature significantly but improved confidence in fitted light response curves, especially for situations with higher flux variability.

## Introduction

Plant and soil fluxes play an important role in global carbon (C) cycling and measuring terrestrial C fluxes accurately is crucial for assessing the net carbon balance, especially in relation to management and climate change impacts. Vast amounts of carbon are stored in the soils of northern peatlands (Gorham 1991), and as such they play a vital role in the global carbon cycle (Yu 2011). However, their small but persistent sink strength, which is affected both by climate and management (Loisel et al. 2020), is of importance to many regions in relation to their carbon budgets. Ombrotrophic blanket peat bogs, of which a large proportion is found in the UK uplands (Bain et al. 2011), are important carbon stores and environmentally sensitive mire types (Lindroth et al. 2007), especially in relation to climate change (Gallego-Sala and Prentice 2013).

Environmental conditions affect both CO_2_ flux component processes of net ecosystem exchange (NEE), that is, affecting photosynthesis gains and respiration losses from plant and soil processes. To make accurate predictions on future carbon budgets and peatlands’ carbon sink versus source strength, it is therefore important to model NEE fluxes over a variety of environmental conditions based on accurate model parameters representing NEE gains and losses. Various techniques are available to measure NEE fluxes, each with advantages but also limitations (Oechel et al. 1998). Whilst eddy covariance (EC) NEE flux tower approaches are frequently used to capture a site’s carbon budget, EC systems measure larger flux ‘footprint’ areas and cannot be easily deployed over smaller experimental treatment areas or patches of different vegetation (Baldocchi et al. 2001). Chamber-based measurements of NEE fluxes are therefore frequently used in plot-scale studies to obtain such parameters (Peichl et al. 2018; Shin et al. 2023) for individual treatments or vegetation types to allow upscaling the manual fluxes over time based on fine resolution (e.g. hourly) climatic data (e.g. temperature and light) to derive integrated carbon budgets (Wieder et al. 2009; Clay et al. 2015; Heinemeyer et al. 2019, 2023; Keightley et al. 2023; Sterk et al. 2023). Such measurements have so far nearly always relied on natural light levels, which can be very limited, especially in upland peatlands with frequently thick cloud cover, mist or fog. However, the upscaling of NEE fluxes especially relies on light response curves by fitting measured fluxes against light levels, which are known to show monthly (Quin et al. 2015) or seasonal differences (Bellisario et al. 1998), and a narrow range limits the confidence in accurate predictions of key fitted parameters such as maximum photosynthetic rates (Peichl et al. 2018). Artificial light has long been used to obtain full light response curves in leaf-scale chambers (e.g. using analysers with leaf or shoot cuvettes, e.g. LI-COR 6800 (2025a)) but is still uncommon in larger-scale chambers placed over larger vegetated surface areas.

To address such light limitations, two recent chamber-based studies reported the use of LED systems (Peichl et al. 2018; Shin et al. 2023), both with large light units placed onto the top of the flux chamber. Such systems are heavy, have considerable power requirements, block out natural light and cause potential changes in environmental chamber conditions (e.g. temperature) with required cooling (Shin et al. 2023), which can limit its use in remote places. There is a clear need to test the use of supplementary light in such places. In this study we aimed to test the use of the latest handheld LED torch technology (IMALENT SR16 (2025)) to provide supplementary light (without blocking out natural light) during in situ chamber NEE flux measurements at a UK upland peatland site as part of an ongoing long-term study assessing climate and vegetation management impacts on ling heather-dominated (i.e. *Calluna vulgaris*) blanket bogs (Heinemeyer et al. 2019). Specifically, NEE measurements were carried out on regrown areas of previously burnt or cut heather areas and on unmanaged, old heather areas. Measurements included different shading levels and fitted light response curves were compared between measurements with or without additional LED light. We expected to observe a visually improved model fit, due to an increased light irradiance range, but hypothesised no significant effect on model parameters and overall model fit, which would confirm that light response curves under limited light conditions are robust and can be trusted even when visual inspection would suggest otherwise. We also provide additional tests on light characteristics and chamber temperature changes in relation to light source distance, irradiance levels and measurement time.

## Materials and methods

### Site description

The study site, Whitendale, is an upland blanket bog peatland in the Forest of Bowland in Lancashire in the northwest of England, at 53°59′04″ N; 2°30′03″ W (UK Grid Ref SD 672543) about 410 m a.s.l. The site is part of a long-term experiment (Peatland-ES-UK (2025)), with two further blanket bog sites in Northern England. At each site there are two catchments of about 10 ha which had been under historic heather burn management. After one year of pre-management monitoring in 2012, one catchment continued to be under a burn management, whereas the other was allocated an alternative cutting management (*c.f.* pictures in Ashby and Heinemeyer 2021). Moreover, unmanaged (uncut) plots were included as part of a replicated experimental block design of 5 × 5 m monitoring plots for all treatments resulting in four plots each for burnt, cut with or without brash removal and uncut plots at each site. The overall experimental and management design is fully described in two published and peer-reviewed reports (Heinemeyer et al. 2019, 2023), which also provide more detailed information on site climate, soil type, hydrology and vegetation; the following section provides an overview on relevant site condition parameters for Whitendale.

The soil is a poorly drained organic peat (soil series 1011b Winter Hill; Cranfield 2025) with an average peat depth of 1.7 ± 0.4 m at the experimental plots with an average slope of 8 ± 3°. The peat depth across the entire catchment area ranged from 0.2 m to 4.5 m (i.e. with shallower areas on steeper slopes). The mean annual water table depth (during 2012–2022) was − 9.0 ± 6.9 cm at the measurement plots. The study area has no drainage ditches, although several erosion gullies (likely related to historic overgrazing by sheep) are present. The vegetation represents a semi-natural heather-dominated blanket bog with various growth stages of heather (*Calluna vulgaris*) cover in addition to other bog vegetation including mainly cotton-grass (*Eriophorum vaginatum* and *E. angustifolium*) and various *Sphagnum* moss and other moss species. Vegetation is heather-dominated with a mean heather height and cover in mature stands of about 35 cm at 60% and about 25 cm and 35% in the burnt and cut areas (managed in 2013), respectively.

### Abiotic variables

**Fig. 1:**
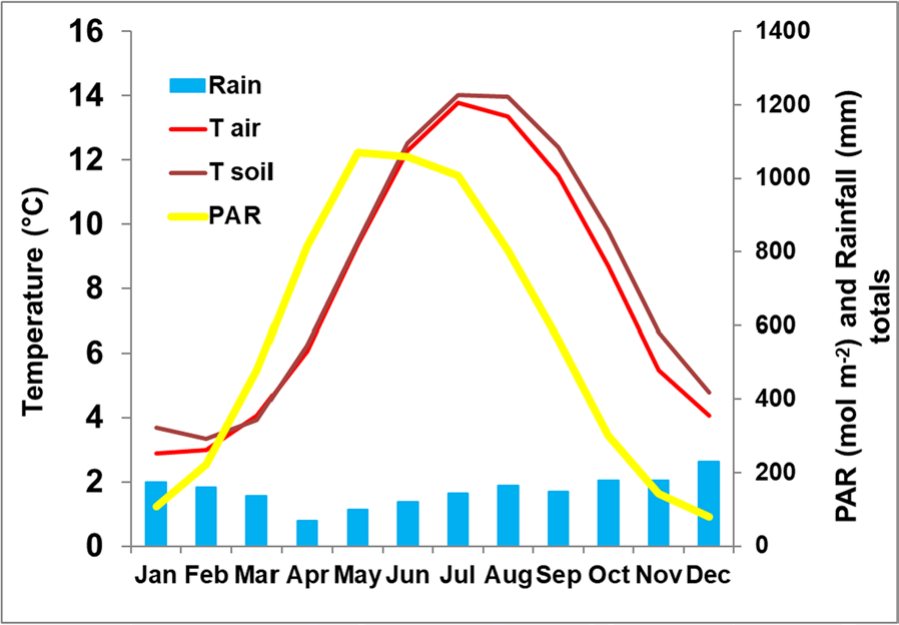
Average monthly climate conditions. Shown are mean monthly temperatures for air (T air) and soil (T soil) and average monthly totals for photosynthetically active radiation (PAR) and rainfall (Rain), at the Whitendale site during 2012–2023 based on hourly measurements

**Fig. 2:**
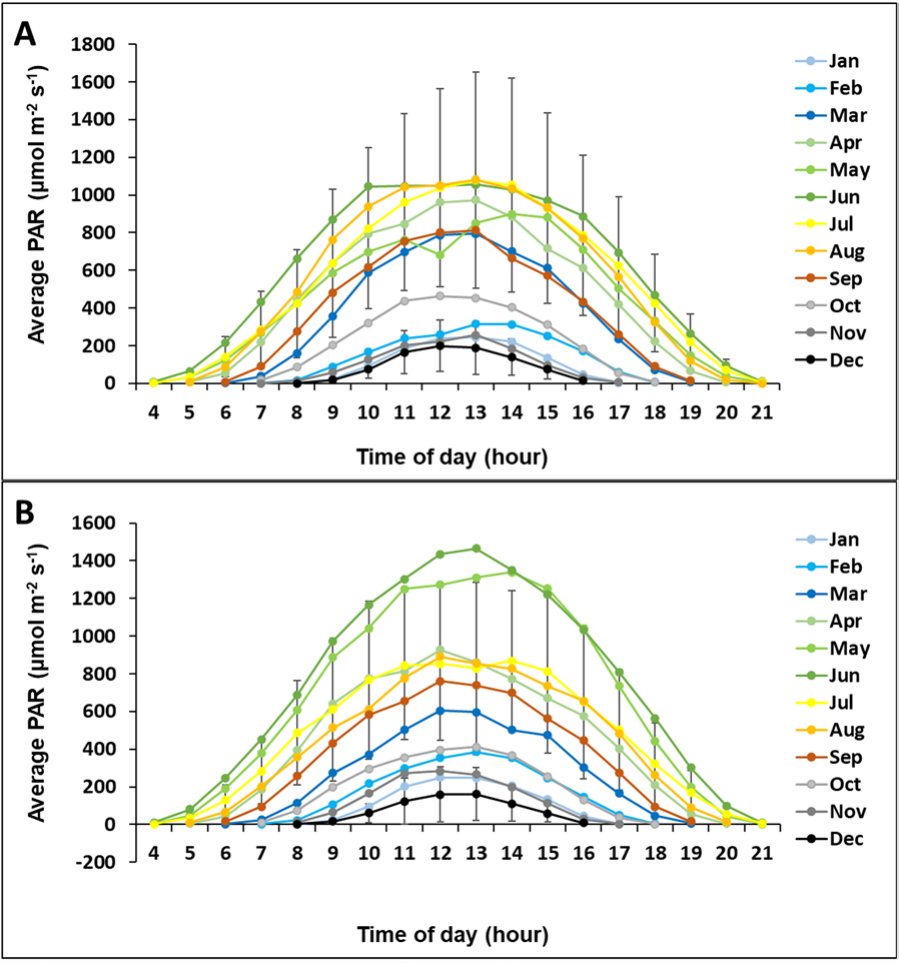
Average daily light conditions per hour of day per month. Hourly average measurements (readings every 30 s) of photosynthetically active radiation (PAR) at Whitendale during **A** 2022 representing an average year versus **B** 2023 with an atypically bright early season (May–June). For clarity, standard deviation is shown only for the months with lowest (December) and highest (July) variability

Climatic data for the field site were obtained using a Skye weather station (Heinemeyer et al. 2023), with hourly mean values for air temperature and photosynthetically active radiation (PAR) being recorded at 2 m height together with total hourly rainfall at ground level and soil temperatures at 5 cm depth. Water tables were measured at monitoring plots at 6-hourly timesteps (Heinemeyer et al. 2019, 2023). The annual mean (± standard deviation during 2012–2022) air temperature was 7.8 ± 0.4 °C and total precipitation was 1795 ± 272 mm during the published ten-year study period, and the average climatic conditions are shown in Fig. 1. The average daily light conditions measured as PAR are generally highest during June to August and lowest in December with noticeably low PAR levels from October until February (as shown for 2022 in Fig. 2A). However, during 2023 May and June were exceptionally bright and autumn was quite dark. The PAR levels in October and November 2023 were similarly low compared to the long-term (i.e. vs 2012–2023) average with a total PAR sum of 283 versus 300 mol m^−2^ and 152 versus 142 mol m^−2^, respectively. PAR, air and soil temperatures were also measured during flux measurements using sensors attached to the gas analyser.

### CO_2_ flux measurements

Flux measurements were performed in the field at the Whitendale site (since 2012) on permanent plots over a 30 cm diameter area marked for NEE flux measurements and followed published methods (Heinemeyer et al. 2019). Fluxes reported in this study were measured on 23 October 2023 as outlined below. Vegetation within the flux areas included mainly a combination of heather, cotton-grass, *Sphagnum* moss (mainly *S. pallustre, papillosum* and *subnitens*) and other moss species (mainly *Hypnum jutlandicum*).

**Fig. 3:**
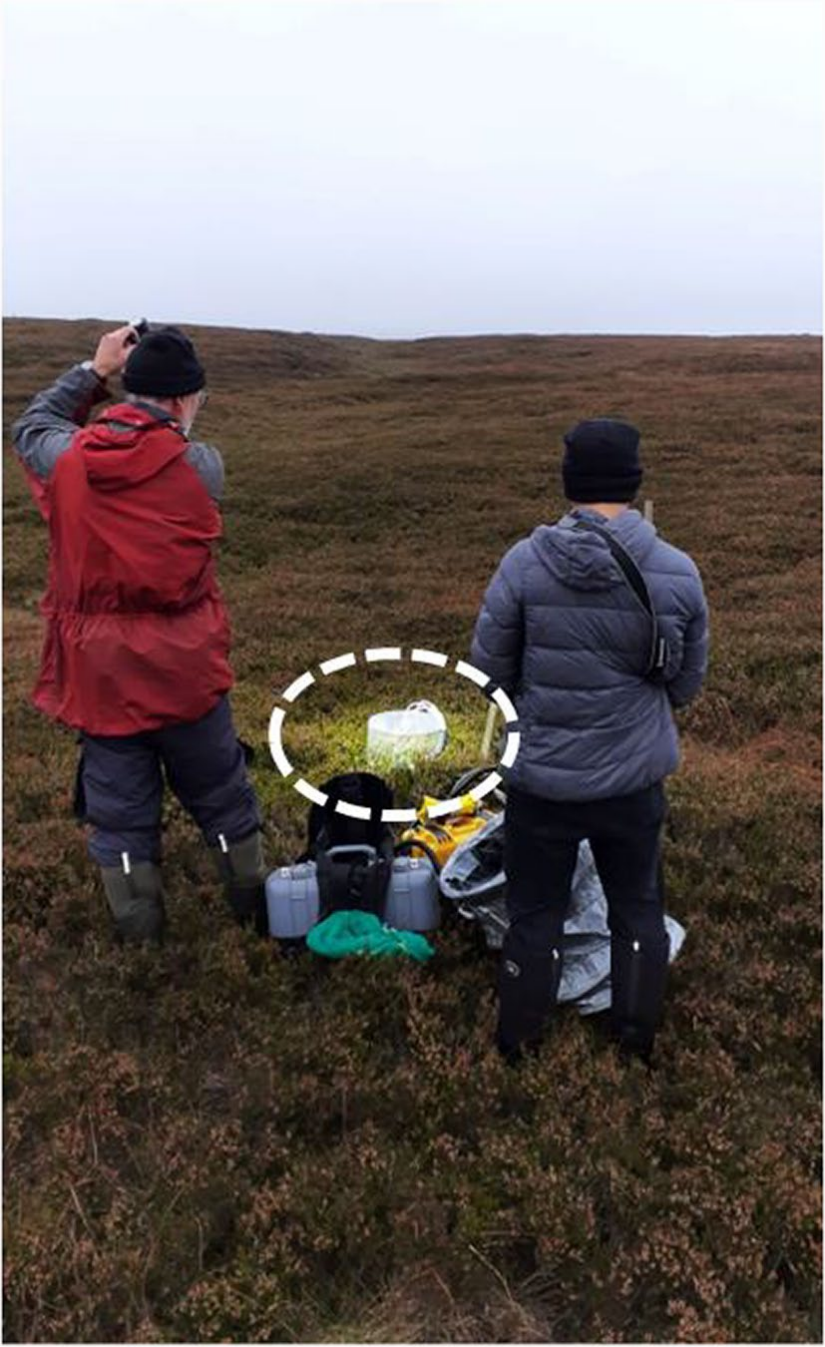
Deployment of the LED torch during net ecosystem exchange (NEE) measurements. Flux measurements were taken with an acrylic chamber on a blanket bog peatland site on 23 October 2023 during cloudy conditions with frequent mist and in fog. The IMALENT SR16 torch illuminated a circular area of about 1 m diameter (see dashed white outline) when held at a distance of about 1.5 m at a slight angle above the NEE flux chamber. Note the shade mesh (green) and the dark cover (silver) used to alter ambient light conditions to obtain a range of light conditions for light response curve modelling. The picture was taken under very cloudy conditions at Nidderdale in North Yorkshire, a similar blanket bog site to Whitendale

A custom-built clear acrylic chamber (Biology Mechanical Workshop, University of York, UK) with an internal diameter (i.e. surface area) of 29.5 cm and a height of 31 cm (about 22 L), connected to an annually cross-calibrated and a few months previously factory calibrated infrared gas analyser (IRGA; Model 8100A, LI-COR, Lincoln, NE, USA), was used for NEE flux measurements (see Fig. 3). Air temperature (sensor: Therm 30 K OHM@25C, part number: 434-08943, LI-COR, Lincoln, NE, USA), soil temperature (Type E thermocouple, part number: 8100-201; LI-COR, Lincoln, NE, USA) and  PAR (sensor: QS5—PAR Quantum Sensor, Delta-T Devices, Cambridge, UK) were recorded (every second) at the top inside of the flux chamber. On plots where the vegetation was taller than 30 cm (i.e. all unmanaged, mature heather plots), an extra 33 cm tall collar of the same material was sealed under the flux chamber using clear tape (about 45 L). A pressure vent at the top of the chamber avoided over-pressuring during chamber placement. No soil collars were used as this alters soil fluxes (Heinemeyer et al. 2011) due to cut roots (causing decomposition) and peat hydrology (causing pooling); the chamber base was instead sealed on the outside during the measurement by means of wet *Sphagnum* moss when required.

The CO_2_ concentration within the chamber was measured every second (s) for 45 s per light level (analyser noise < 1 ppm). Different light levels were achieved by using an LED torch (IMALENT, SR16, 55,000 lm; Guangdong, China) which utilises 16 American CREE XHP50.3 HI LEDs (for further specifications see IMALENT SR16 (2025)) and two horticultural green shade meshes (light and dark). Measurements were done in the order of LED light, dark cover, dark and light shade mesh and natural light (missing if only natural light was measured at the start). This order, together with the short measurement periods, ensured that chamber CO_2_ levels stayed close to ambient CO_2_ conditions (± 20 ppm; normally about 380–420 ppm). For maximum light, the LED torch was held facing the sun's position at a slight angle (~ 30–40°) and about 1.5 m distance to the flux chamber, which illuminated an area of about 1 m diameter (see Fig. 3). The LED torch was switched on about 5 s before the flux measurement recording started. The two shading meshes were placed sequentially over the chamber (reducing natural PAR by about 45% and 80%). For the dark measurement, a custom-made dark cover was placed over the chamber, blocking out all light and therefore measuring ecosystem respiration (*R*_eco_). The acrylic material reduced the PAR inside the chamber by 10% (Heinemeyer et al. 2014, 2019). However, three replicates of the mown with and without brash removal were not measured with the dark shade mesh (S2).

The LI-COR Viewer software (v3.2.2; see LI-COR 8100 (2025b)) was used to derive the CO_2_ fluxes from the most linear portion of each light level measurement (this was mostly the last 30 s), with PAR levels averaged over that period. For mature heather plots, an estimated plant volume was subtracted from the chamber volume (Morton and Heinemeyer 2018). All CO_2_ concentrations were corrected for chamber temperature, and pressure and fluxes were derived using chamber volume and collar surface area and are expressed in µmol CO_2_ m^−2^ s^−1^.

For each measurement date, a LRC was modelled following published examples (Brown 2001) as nonlinear (hyperbolic) curve fit for all combined NEE flux replicates (i.e. not per individual plot replicate) either for the overall combined or separate heather managements (*n* = 4), using the equation:

1 $${\text{CO}}_{2} {\text{flux}} = \left( {P_{{{\text{max}}}} \times {\text{PAR}} / \left( {{\text{PAR}} + {\text{K}}_{{\text{m}}} } \right)} \right) + R_{{{\text{eco}}}}$$

where CO_2_ flux is the modelled CO_2_ flux at a particular light level, *P*_max_ is the maximum CO_2_ uptake of the curve, PAR is the amount of light in µmol m^−2^ s^−1^, K_m_ is a calculated constant (i.e. PAR level at half *P*_max_) and *R*_eco_ is the modelled maximum CO_2_ release (equivalent to *R*_eco_ in the dark). Following Brown (2001), *P*_max_, *K*_m_ and *R*_eco_ were calculated using the Solver function in Excel (Microsoft Office 365; version 2407; Build 16.0.17830.20210), which was set to minimise the variability of the modelled curve fit through the measured data points (see Fig. 6 and 8). The slope at KM (PAR/(PAR + KM)) determines the shape of the curve fit and is termed PAR-slope. Light compensation points were calculated from this equation by solving the equation for PAR and setting NEE to zero (i.e. PAR level where carbon uptake and release are equal).

### LED torch assessment

Wavelength spectra were assessed with an Ocean Insight FLAME-T-UV-VIS-ES Spectrometer (Serial No FLMT08252) calibrated for absolute irradiance. Measurements were based on an average of 5 scans, with an integration time of 18 ms. Assessment of PAR levels was done using a Skye PAR Quantum (SKP215/SS2) connected to a SpectroSense2 metre (SKL904/1). Both were calibrated (03/03/2022) against a UKAS calibrated metre and National Physical Laboratory UK reference standard lamp with an uncertainty of < 5%.

The PAR levels across an area were assessed in the laboratory by shining the torch at a similar angle to that in the field over a 1 × 1 m grid fixed to the floor (with 88 locations marked onto large sheets of paper, with distances becoming narrower towards the centre; see Fig. 5B), simulating the torch operation in the field. Light levels were recorded with the handheld Skye PAR meter and projected as a graph using an online tool (VISJS (2025)).

The temperature change inside the acrylic chamber was also assessed in the laboratory (Fig. 7B), considering different PAR (irradiance) levels (400, 800 and 1600 µmol m^−2^ s^−1^) at three different distances (30, 50 and 100 cm) and over four different measurement periods (10, 30, 60 and 120 s).

### Data analysis

Data were processed either with LI-COR instrument software or in Excel. Statistical analysis was performed in R Core Team (2024). For testing chamber temperatures, nonparametric tests were used as the data were non-normal even after transformation and the number of replicates was very low for light response curve parameters (comparing LED to natural light overall across the four managements; *n* = 4). For chamber temperatures, a Kruskal–Wallis test was used and for light response curve parameters a dependent *t* test (paired-samples). Light response curve models were compared with individual parameters tested using a nonparametric Wilcoxon rank test (wilcox.test); the overall model fit (over the respective PAR ranges) was compared based on model performance based on root mean square error (RMSE) and based on the p-values of deviations (raw value minus predicted value) between both models (over the total PAR range) as calculated by either a Wilcoxon rank test or a parametric *t* test (when data were normal distributed).

## Results

The monthly climate conditions at Whitendale are typical for British upland blanket bogs, with fairly high rainfall throughout the year, mostly during autumn and winter (Fig. 1). Light conditions (i.e. PAR) peaked in early summer, with very low light levels during October to February, and temperatures peaked in late summer, with soil temperatures at 5 cm depth being slightly and consistently warmer than air temperature (Fig. 1). The average daily PAR light levels are generally highest during June to August and lowest in December with noticeably low mean PAR levels from October until February (as shown for 2022 in Fig. 2A). However, during 2023 May and June were exceptionally bright and autumn was quite dark (Fig. 2B); during October and November 2023 the total PAR sum was only 283 and 152 mol m^−2^, respectively.

The field testing of the supplementary LED light was conducted during a low light level period in autumn of 2023. The setup at Whitendale was similar to the one shown in Fig. 3. The LED light illuminated a large area (about 50–100 cm in diameter) at a distance of about 1.5 m, and the PAR level (irradiance) was visually higher than natural levels (Fig. 3). PAR levels were monitored with a computer (every second) to provide verbal feedback to the person directing the beam of light (to allow small adjustments to ensure similar and fairly stable PAR levels for all LED flux measurements; however, PAR levels are naturally variable and are averaged across a stable flux measurement period). Measurements were conducted over different heather management areas consisting of no management (~ 30-year-old heather) versus managed (in 2013) heather by either burning or cutting (with or without leaving brash).

**Fig. 4:**
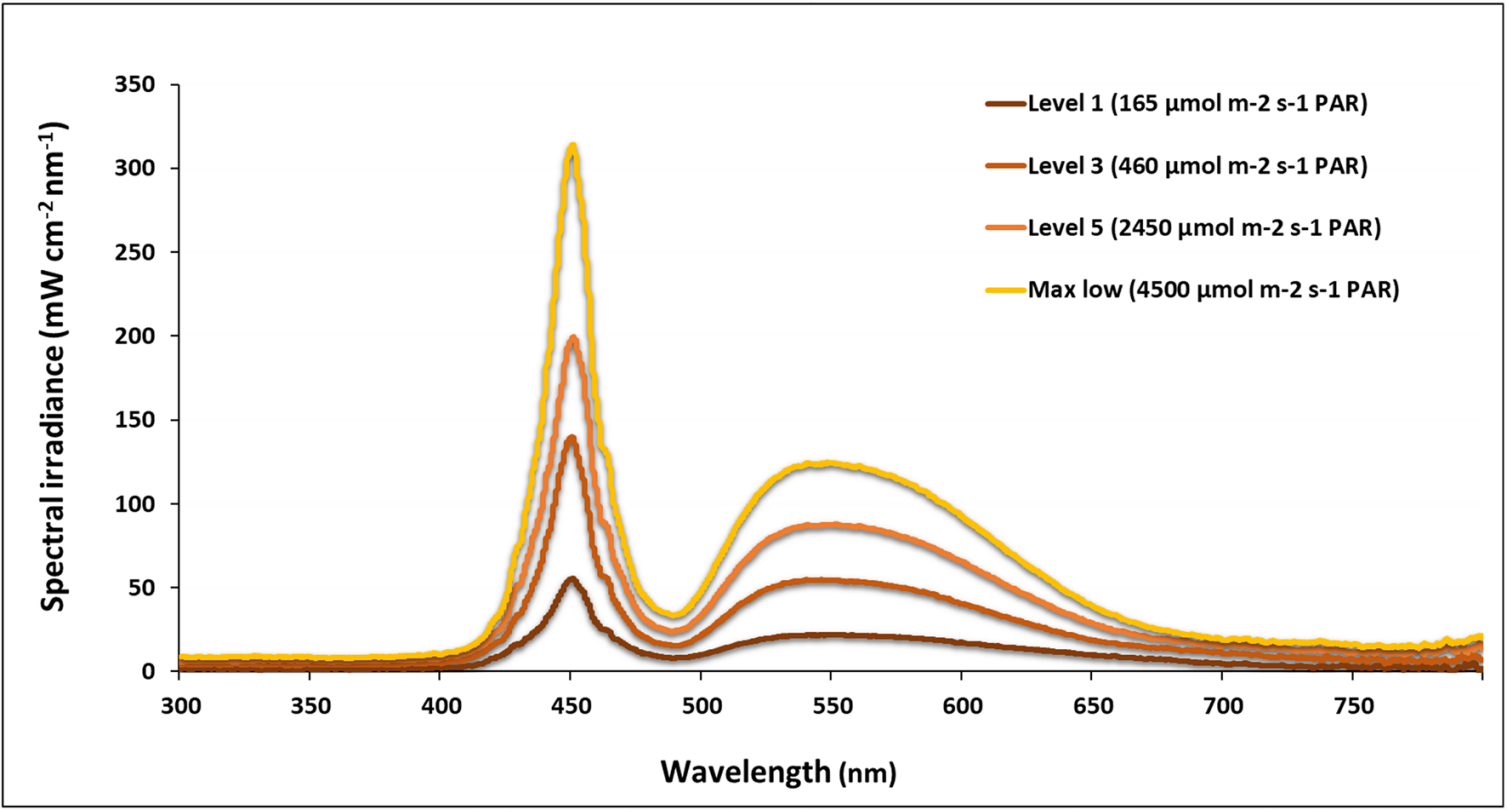
Spectral irradiance for the LED torch (IMALENT SR16). Wavelengths were measured over a range of 300–800 nm at 1.5 m above the spectrum sensor for 4 different light output settings (i.e. levels 1, 3, 5 and max low with their corresponding photosynthetically active radiation (PAR) in µmol m^−2^ s^−1^). The low maximum (Max) value reflected a decline in output irradiance over the period of deployment at maximum light irradiance

To assess the LED light quantity and quality, the torch was assessed in the laboratory with a spectrometer and a PAR quantum sensor (also at a 1.5 m distance). The spectral analysis (Fig. 4) revealed two peaks, a narrow one at 450 nm and a broad one between 500 and 650 nm within the overall measured range from 300 to 800 nm, and these peaks remained constant when changing light levels.

The assessment of PAR levels (irradiance) with a quantum sensor revealed a very high range of PAR output across the various torch settings (Table 1), which covered the observed monthly range of average hourly PAR levels at 13:00 GMT in the field. Notably, the user can toggle between different light levels although the deployment of the maximum output is restricted by the battery voltage. The highest PAR output irradiance (after running it for 30 s) was recorded as about 4500 µmol m^−2^ s^−1^, which is far greater than any natural mean hourly PAR level (Fig. 2), and the two lower levels (settings 4 and 5) cover most field observed mean hourly PAR ranges. However, the light output at the higher levels drops with deployment time (due to heat) and thus short periods of high supplementary light levels are a feature of this LED torch.

**Table 1:** Comparison of photosynthetically active radiation (PAR) in the laboratory and field

Measured in the laboratory		Measured in the field			
LED Irradiance	PAR (µmol m^−2^ s^−1^)	Month	PAR (µmol m^−2^ s^−1^)	Month	PAR (µmol m^−2^ s^−1^)
1	165	January	245/248	December	188/161
2	335	February	315/385	November	257/266
3	460	March	796/597	October	454/413
4	1050	April	974/861	September	812/739
5	2450	May	852/1311	August	1082/856
Max	4500	June	1055/1465	July	1078/829

PAR was measured at 1.5 m below the LED torch (IMALENT SR16) for different light irradiance settings using a PAR sensor in the laboratory and is compared to field-measured monthly PAR level ranges. The maximum light level was measured in the laboratory after 30 s (i.e. slightly lower than the actual maximum value but more stable due to an initial declining in light irradiance), and the average hourly PAR levels are reported for measurements taken by an hourly weather station at 13:00 for January to December in the field at the measurement site (Whitendale) in 2022/2023

**Fig. 5:**
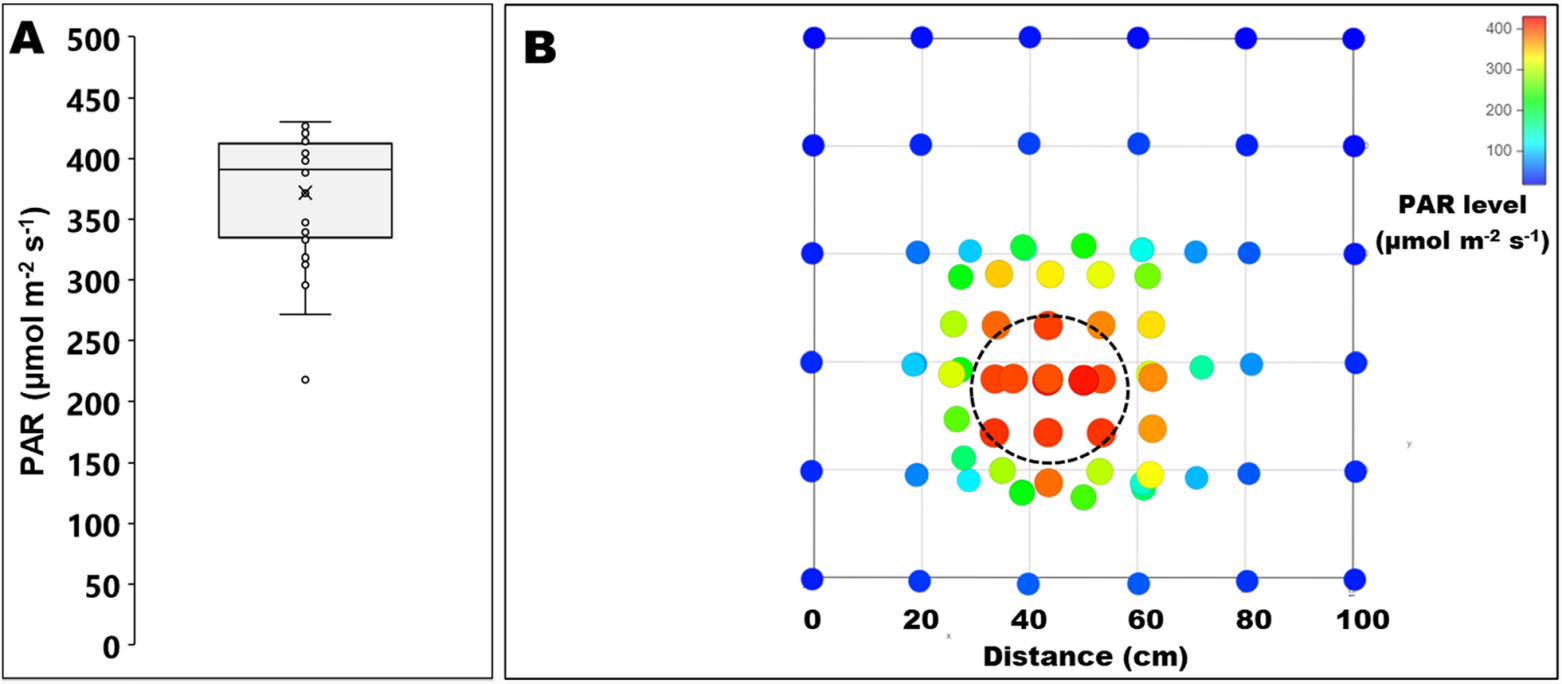
Light levels of photosynthetically active radiation (PAR) measured in the lab. PAR was measured at 1.5 m distance from the flashlight (held at a similar angle to that in the field but without a chamber) at 34 points inside the projected 30 cm diameter chamber area shown in a box plot **A** with the median (line) and average (cross) PAR levels (with minimum, maximum, median, first quartile, and third quartile) and **B** across a wider 100 cm × 100 cm grid area (dashed line indicating the chamber area) with a further 58 points (note: several dots at the centre overlap)

Further tests in the laboratory assessed the PAR levels of the light cone area in relation to the 30 cm diameter chamber area, which revealed a very similar PAR level across the chamber area measured at a distance of 1.5 m with a mean of 370.9 µmol m^−2^ s^−1^ ± standard error of 9.3 µmol m^−2^ s^−1^ for the 34 points within the chamber area (Fig. 5A), but a noticeable drop in PAR levels beyond the 50 cm diameter central light cone area (Fig. 5B).

**Fig. 6:**
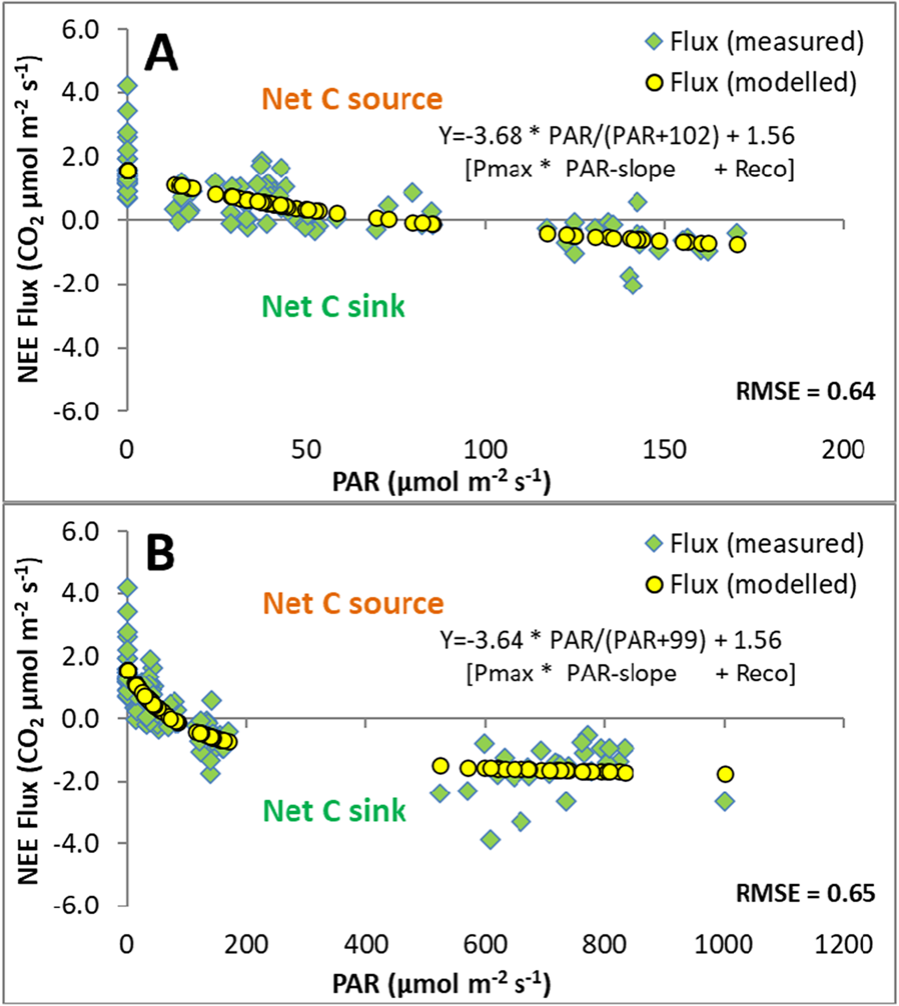
Net ecosystem exchange (NEE) CO_2_ flux comparison of measured versus modelled fluxes. Measured NEE fluxes (green diamonds) from 28 plots were combined for different heather management replicates (*n* = 8 burnt, 16 mown, 4 uncut). NEE fluxes were obtained (measured) under ambient light with sequential shading **A** and with additional supplementary LED light **B**. Modelled values (yellow circles) respond to fitted light response curves (modelled; see equation parameters). Negative values indicate a net carbon C sink, positive a C source. Root mean square errors (RMSE) are shown indicating model fit performance. Note the visually improved confidence in the model fit in **B** versus **A** due to the extended *x*-axis PAR range

On 23 October at Whitendale, the natural PAR levels during the CO_2_ flux measurement period (9:00–13:00 GMT) were less than 180 µmol m^−2^ s^−1^. The supplementary LED light was set to increase the PAR level to between 500 and 1000 µmol m^−2^ s^−1^ (Fig. 6), which provided a much larger range for applying a model fit to obtain light response curve (LRC) parameters but, importantly, fitted model parameters were near identical.

**Fig. 7:**
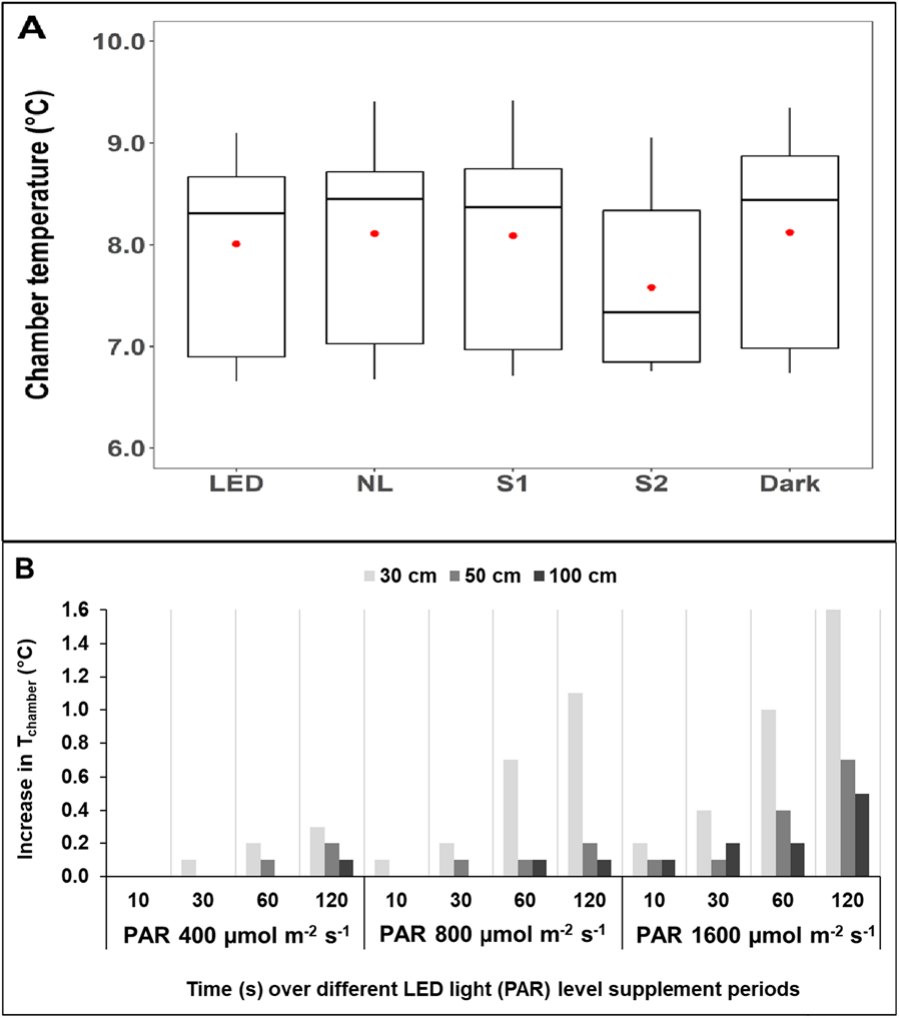
Air temperature measured inside the carbon flux chamber (*T*_chamber_) during CO_2_ measurements. **A** box plots with the median (black line) and average (red dot) chamber temperatures (with minimum, maximum, median, first quartile, and third quartile) during field measurements across all 28 plots comparing periods of supplementary light (LED), natural light (NL), light shade mesh (S1), dark shade mesh (S2) and dark (Dark) measurements (each over 45 s). The number of replicates was *n* = 28 each but for the S2 treatment (*n* = 16). **B** Temperature increases inside the flux chamber during laboratory trials testing three different irradiance settings on the torch responding to different photosynthetically active radiation (PAR) levels (400, 800 and 1600 µmol m^−2^ s^−1^) at three different distances to the light source (30, 50 and 100 cm) and across four different measurement periods (10, 30, 60 and 120 s). Differences in temperature between repeated measurements (*n* = 3) for each combination could not be detected as they were less than 0.1 °C (the recorded measurement accuracy of the LI-COR chamber temperature sensor)

Moreover, the about 1.5 m distance of the LED source to the chamber prevented artificial heating of the inside of the chambers when compared to temperatures of the various natural light and shading levels (Fig. 7A), and there was no statistically significant difference in chamber temperatures between the five light levels (*H*(4) = 4.9861; df = 4; *p* = 0.2887; *n* = 128) between the five light treatments (*p* = 0.289). The slightly lower mean and median temperatures in the dark shade treatment were due to fewer measurements during warmer periods (*n* = 16 versus *n* = 28 for all other light levels). We also tested for any potential temperature increase inside the chamber under controlled laboratory conditions, testing various irradiance levels, distance to the chamber and time periods (Fig. 7B). Whilst there was an overall increase in chamber temperatures with decreasing distance and increasing irradiance and light period, the changes were overall very small. Particularly for the field operational levels (about 1.5 m, < 800 µmol m^−2^ s^−1^ and ~ 30 s), the increase was less than 0.3 °C. However, for closer or longer flux periods impacts could be important.

**Fig. 8:**
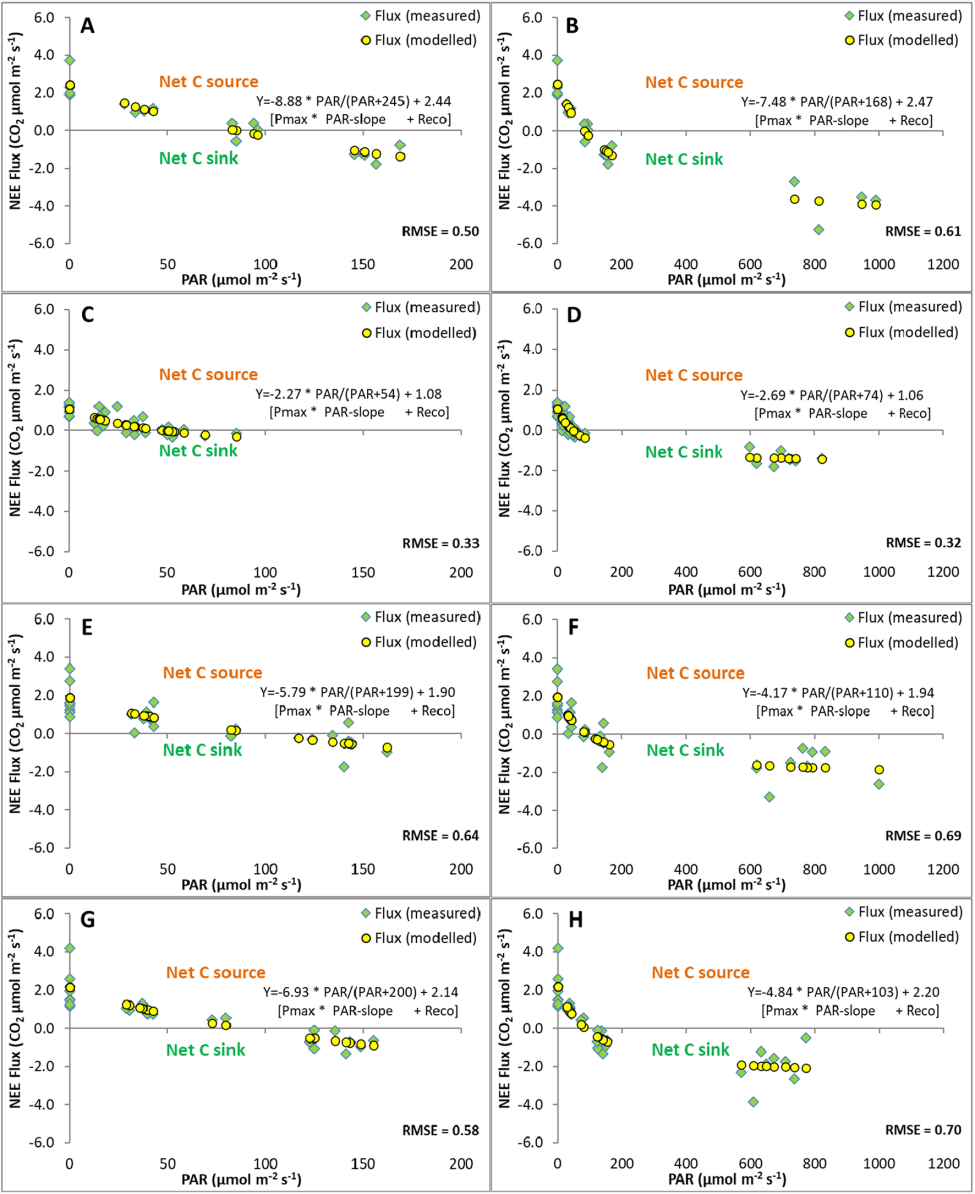
Net ecosystem exchange (NEE) CO_2_ flux comparison of measured versus modelled fluxes for individual heather managements. NEE fluxes from 28 plots were measured (green diamonds) under ambient light with sequential shading **A**, **C**, **E**, **G** and with additional supplementary LED light **B**, **D**, **F**, **H** for different managements with individual number of replicates, with unmanaged, mature heather (A, B: *n* = 4) versus 10-year-old heather on burnt (C, D: *n* = 8) or mown plots with brash left and with brash removed (E, F and G, H, respectively: *n* = 8). Modelled values (yellow circles) respond to fitted light response curves (modelled; see equation parameters) for maximum NEE (Pmax), light response (PAR-slope) and ecosystem respiration (*R*_eco_). Negative values indicate a net carbon (C) sink and positive values a C source. Root mean square errors (RMSE) are shown indicating model fit performance. Note, for example, the visually improved model fit in **B** versus **A** due to the extended x-axis PAR range

The NEE flux measurements under natural light conditions and various shading levels provided a much narrower range of flux versus PAR levels (Fig. 6A) compared to that with supplementary LED light (Fig. 6B). Low light levels, such as those on the measurement day during misty and foggy conditions, are typical for UK blanket bogs in autumn and showed typical LRC behaviour changing from positive NEE fluxes (net emission) in the dark to negative fluxes (net uptake) in full light. The LRC model fit provided parameters to obtain values for maximum photosynthetic uptake (Pmax), light sensitivity of the slope (KM; PAR-slope) and ecosystem respiration (*R*_eco_) in the dark, (see Eq. 1 in the methods). This model also allows the calculation of light compensation points where the modelled NEE flux is zero (i.e. where the fitted LRC crosses the x-axis), which were less than 100 µmol m^−2^ s^−1^ for the measurements at the various heather management plots under the natural light conditions (Fig. 8A, C, E, G).

The supplementary LED light extended the maximum PAR range on the management plots from about 100-200 (Fig. 8A, C, E, G) to mostly about 600–800 µmol m^−2^ s^−1^ (Fig. 8B, D, F, H), which visually improved the LRC fit far above the light compensation point (LCP; i.e. PAR level where *y* = 0) and towards PAR levels much nearer Pmax. However, whilst LRC parameters for Pmax and PAR-slope changed slightly these changes were not significant (Table 2) and values for Reco hardly changed, which confirmed our hypothesis in relation to a visually improved LRC fit without affecting the overall model. LRC predicted values for Pmax decreased on average by 1.17 µmol m^−2^ s^−1^ when fitted to natural versus LED light conditions and the PAR-slope values decreased by 61. Only the LRC for Pmax and PAR-slope on burnt plots (Fig. 8C, D) increased, which was the least light responsive management under natural light.

**Table 2:** Statistical comparisons of light response curve parameters

**A**) Overall light response curve parameter comparison			
Parameter	*n*	*W*-value	*p*-value
LCP	8	6	0.665
PAR-slope	8	4	0.312
Reco	8	9	0.885
Pmax	8	10	0.665

**B**) Individual light response curve model data comparison			
Management	*n*	*W*- or *T*-value	*p*-value
ALL	248	7626 (*W*)	0.913
DN	40	0.785 (*T*)	0.437
FI	80	0.920 (*T*)	0.361
LB	68	− 1.250 (*T*)	0.216
BR	68	504 (*W*)	0.367

Tests were based on either a Wilcoxon rank test (*W*) or a *T* test (*T*) for the overall light response curve model parameters (LCP = light compensation point, PAR-slope, ecosystem respiration = Reco, maximum photosynthetic uptake = *P*_max_; df = 3) and the individual model data for the with and without LED flashlight supplement measurements (df = 1), showing sample number (*n*), test (*W* or *T*) and *p*-values. For **A**) the individual model parameters for each management were considered (four pairs or replicates per light treatment). For **B**) flux data were assessed comparing groups of either all managements combined (ALL) or individually (DN, unmanaged; FI, burnt; LB and BR, mown with either brash left or brash removed, respectively)

The modelled NEE for the combined (all managements) fluxes for without versus with supplementary LED light measurements did not differ significantly for Reco, Pmax and PAR-slope parameters (Table 2A) with very little difference between model parameter values (Fig. 6). Similarly, the individual managements did not show any significant difference in their model fit (Table 2B) or any meaningful change in their LCP or the LRC parameters (Table 2A) although the combination of replicates for light response curves per management limited the statistical test options), and the reduction in LCP with supplementary LED light for the uncut, burnt, mown with brash and mown with brash removed management was less than 3% of PAR (i.e. reduced by 2.1, 0.6, 1.9 and 3.8 µmol m^−2^ s^−1^, respectively). Moreover, the model fit as measured by the RMSE between with and without additional LED light was nearly identical for all managements combined (Fig. 6) and very similar for individual managements (Fig. 8).

## Discussion

Chamber-based ecosystem carbon fluxes are frequently used to determine the carbon balance over low vegetation, for example, in relation to vegetation cover (Li et al. 2008; Poyatos et al. 2014; Morton and Heinemeyer 2018), climate change (Kim et al. 2006) and management (Muhr et al. 2011; Heinemeyer et al. 2019). Here we report on a practical advance in seasonal field monitoring of manual chamber-based carbon fluxes in low vegetation ecosystems, where light conditions are often limiting. The IMALENT SR16 LED torch proved a very versatile device for NEE flux measurement in a remote upland peatland under very low light conditions in autumn. Although the handheld LED deployment resulted in more variable light levels reflecting slight changes in distance and angle, compared to two previous studies using fixed LED covers (Peichl et al. 2018; Shin et al. 2023), user interrogation of light levels in real time facilitated a fairly tight light range between measurements (Fig. 6) with a quite even PAR level across the chamber area (Fig. 5). The previous studies (Peichl et al. 2018; Shin et al. 2023) used plant growth LED lights fixed on top of the chamber, different to natural light, but with a wider overall second wavelength peak range aligned with both photosynthesis absorption maxima compared to the IMALENT torch. The torch output was also different to natural daylight although the two peaks at 450 and > 550 nm fall within the two main areas of plant photosynthesis (i.e. chlorophyll a and b) as previously shown (Shin et al. 2023). However, the torch supplemented photosynthetically relevant light levels to the natural light range (as it was not fixed to the top of the chamber natural light still illuminated the chamber), and future LED torches could optimise the wavelength match, especially for the second peak to be higher and around 650 nm. Moreover, any spectral changes due to variation in light angles should be less important due to the additional background of diffuse natural light during the cloudy/misty conditions and the plant-relevant PAR levels being measured inside the chamber. Whilst a tripod could be considered, this would need to be heavy duty and would likely cause weight issues for remote and manual field work. Importantly, the artificial LED light from this torch requires no additional power for cooling between the LED light and the chamber (although it operates an internal fan during higher light output), as is the case for top-mounted LED lights based on plant growth lights (Peichl et al. 2018; Shin et al. 2023). Such heavy and power intensive top-mounted LED chamber systems are therefore less portable (Shin et al. 2023) and ill-suited for remote field deployment, where the small handheld torch offers a significant advantage. Our short flux measurement periods also prevented any significant heating inside the chamber from the handheld torch, although longer measurement periods with higher light intensities were shown to have the potential to considerably affect chamber temperatures (Fig. 7B). However, the internal battery power limited total deployment time at the ‘High’ output setting to about 30 min (with about 50 s of LED light per NEE flux sequence this equates to just under 40 LED supplemented flux measurements), but small supplementary batteries or additional units could easily extend the deployment time and ‘High’ output levels.

**Fig. 9:**
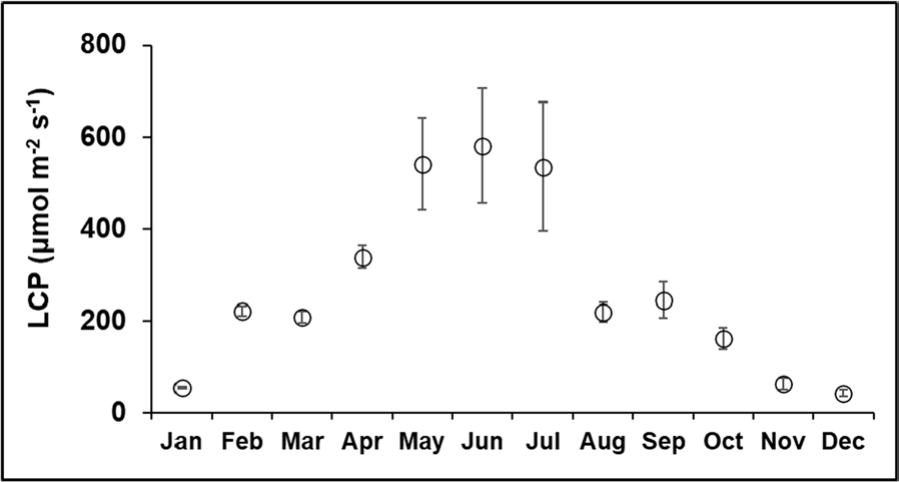
Average (± standard deviation) light compensation points (LCP) per month during 2012–2022. The monthly light levels as photosynthetically active radiation (in µmol m^−2^ s^−1^) for zero net ecosystem exchange CO_2_ flux were estimated based on 44 seasonal light response curves for heather-dominated vegetation at Whitendale and two other similar blanket bog sites (data from Heinemeyer et al. 2023)

The very low weight (about 1300 g including the battery) and low power consumption of the handheld LED torch offer a very flexible approach for field flux measurements. Low weight is especially important as it allows the user to regulate the light levels in the chamber by manually adjusting the distance to the torch based on feedback from real-time light readings from laptop wireless monitoring (every 1 s). Moreover, the various output settings on the torch allow setting the maximum output to be slightly above the expected seasonal (i.e. monthly) maximum light (i.e. PAR) level at a site (e.g. in our autumn flux study about 450 µmol m^−2^ s^−1^ as shown in Table 1). However, the light output for the maximum setting drops somewhat with deployment length (reflecting heat constraints on LED output) and will therefore be more similar for short measurement times such as in our study. In any case, PAR levels were recorded in the field at the same frequency inside the chamber alongside CO_2_ concentrations, which are used to compute fluxes versus the mean PAR levels during that period, which showed very stable PAR levels over the 30 s period. The powerful maximum light output and flexible settings of different output levels of the IMALENT SR16 (Table 1) offer an ideal tool for seasonal and targeted flux measurements with supplementary light. Depending on only natural light levels often limits measuring fluxes near or just above the light compensation point of NEE fluxes (see Fig. 6A). This limitation results in a visually questionable model fit of the LRC and its parameters. Extending the range of net carbon flux uptake measurements by increasing light levels therefore allows the user to obtain a more robust light response curve fit across the C sink versus C source range (see Fig. 6B). The additional LED light allows the extension of the flux measurement range well above the light compensation points, which change seasonally for temperate ecosystems such as observed for this and another two heather-dominated peatlands (see Fig. 9, based on additional long-term flux measurement data (Heinemeyer et al. 2023). This is important even in normally brighter seasons, as cloudy or foggy conditions can severely reduce light levels, especially on mountains, as seen in the lower bound of the standard deviation range in monthly light (i.e. PAR) levels shown for Whitendale in July 2022 (Fig. 2A), to below the corresponding seasonal LCP (Fig. 9).

It is important that our comparison shows that, whilst the supplementary light does improve the visual confidence in the robustness of the LRC model fit, it does not change the model parameters significantly (Table 2). Whilst this implies that using an LED supplementary light is unnecessary, this finding is important and reassuring as otherwise this would question the reliability of many previously published light response curves based on light-limited manual chamber flux measurements. The light response curve fit with supplementary LED light was visually improved (i.e. the extended x-axis PAR range resulted in a more extended curve and therefore visually aided confidence in the model curve fit) and affected some model parameters for the individual management LRCs. Whilst Reco remained largely unaffected, both Pmax and the initial PAR-slope were slightly reduced for all but the burnt management plots, revealing a steeper initial slope and less negative Pmax (Fig. 8). Both parameters will affect modelling NEE fluxes using, for example, hourly light levels, increasing predicted net carbon uptake for lower whilst reducing it for higher light levels. However, when fluxes were combined across all managements, Reco, Pmax and PAR-slope parameters did not differ significantly between with and without supplementary LED light measurements. Similarly, the individual managements did not show any meaningful change in their LCP, and the non-significant reduction in LCP with supplementary LED light was less than 3%. Both assessments, together with the very similar RMSEs for both with and without supplementary LED light; Figs. 6, 8), provided additional confidence in the robustness of the light response curve fit, which could be questioned based on visual assessment of the smaller model fitted curve range under natural light constraints. However, the number of replications was limited in this study and further tests are recommended.

The overall net impact of the improved LED light response curve parameters on cumulative NEE fluxes (e.g. as part of calculating a carbon balance) will depend on the overall light conditions during the modelled time period but seems to be mostly affected at the less frequent higher, near daytime peak light levels, due to a lower Pmax.

These seasonal differences in light response curves, LCP and model parameters and their response to supplementary LED light are of importance to many studies as previously shown for Pmax (Peichl et al. 2018) and other fitted model parameters (Li et al. 2008). Frequently NEE fluxes are measured seasonally or even monthly. However, whilst seasonality in light response curve parameters was considered important as early as 1998 (Bellisario et al. 1998), light (and sometimes also other environmental) response curves are often fitted annually across all measured fluxes (Riutta et al. 2007; Shaver et al. 2007; Wieder et al. 2009; Clay et al. 2015; Keightley et al. 2023; Sterk et al. 2023). Such an overall annual fit approach misses important seasonal differences in LCP (as shown for monthly LCP changes in Fig. 9) and impacts on net carbon sink to source calculation (Heinemeyer et al. 2019) and affects the subsequent annual carbon balance, which further emphasises the need for an in-depth understanding of phenology responses to abiotic conditions associated with climatic changes (Peichl et al. 2018). However, some studies did not consider light responses at all and upscaled NEE fluxes based on other environmental factors such as temperature and water table depth (Goud et al. 2017). We propose that LED lights could become a regular feature for field-based NEE flux measurements over low vegetation in environments with seasonal or local low light conditions, which will substantially improve confidence in seasonal light response curves and therefore annual estimates of the ecosystem carbon balance and linking measured fluxes to model representations and process-level improvements at regional levels (Shaver et al. 2007; Jung et al. 2008).

## Further research

This is the first test of one powerful handheld LED torch at one site, highlighting its potential application and resulting in additional information in relation to advancing chamber-based carbon flux measurements. We recommend further tests of such LED torch light supplemented carbon flux measurements, extending the range of carbon flux uptake by increasing light levels and to do further assessments of comparing obtained light response curves. Tests at further sites across different low vegetation ecosystems and under different environmental conditions will also allow to assess if there are situations where model parameters show meaningful significant differences between LED and no LED supplementary models. Such assessments should also consider the often-overlooked seasonal changes in light response curves as shown here for light compensation points and implications for calculation of an ecosystem’s annual carbon balance.

Whilst the LED torch spectra used in this study differ from natural solar radiation, natural spectra also are not constant; as solar radiation energy irradiated by the sky environment decreases, the solar radiation spectrum also changes (e.g. Siriwardana and Kume 2025). However, further studies should investigate other LED sources and also consider spectra changing aspects like diffuse versus direct radiation (e.g. Wang et al. 2023). Such assessments could also investigate different chamber materials, thickness and light angles affecting the internal light spectra and exploring the use of a tripod.

## Conclusions

This study showcases the application and trial of a handheld LED torch to facilitate field measurements of net ecosystem exchange in heather-dominated upland peatlands. The lightweight and low-power torch addresses flux measurement challenges under limiting natural light conditions in remote areas, which are typical in many upland ecosystems in oceanic and temperate climates with frequent mist, fog and cloud cover especially in seasons with low light levels. The torch successfully increased the PAR levels to well above the light compensation point, determining the PAR threshold for switching from a net carbon source to a net sink, whereas natural light levels were just above this important parameter, questioning the robustness of the overall model fit. Fitted light response curves based on shade responses of fluxes from various heather management treatments revealed an overall good agreement between with and without supplementary LED light. However, the confidence in the model fit was visually much improved with LED light and although ecosystem respiration and the light compensation points remained very similar, the modelled maximum photosynthesis was slightly lower but without any significant differences in the model fit. Our data provide reassurance of the adequate model fit of LRC under limiting light conditions but highlight the overall benefit of improved model fit confidence when using a torch. Future improvements could include the torch LED wavelength spectrum to cover more of the full photosynthetic range and the power supply would benefit from a larger battery or an exchangeable battery port. This supplementary light has the potential to benefit many researchers across the globe, especially where light conditions are seasonally or locally limiting, to obtain more robust seasonal net ecosystem exchange fluxes with ground-based chambers over low vegetation. This study also highlights the importance of considering seasonal changes in light response models as part of modelling overall annual ecosystem carbon balance and net sequestration, for example, in relation to land management and climate change.

## Data Availability

The datasets generated during the presented study are available on reasonable request from the corresponding author.

## Abbreviations

C: Carbon
Reco: Ecosystem respiration
EC: Eddy covariance
LCP: Light compensation point
LRC: Light response curve
Pmax: Maximum photosynthesis
NEE: Net ecosystem exchange
PAR: Photosynthetically active radiation
RMSE: Root mean square error
T: Temperature
